# Pharmacokinetics and Pharmacodynamics of an Antibody Targeting Pathological Tau for the Treatment of Alzheimer’s Disease: Nonclinical Studies in P301S Mice and Cynomolgus Macaques

**DOI:** 10.1002/alz.091630

**Published:** 2025-01-09

**Authors:** Chanchal Sadhu, Wencheng Liu, Joydip Kundu, Jeffery Thompson, Maurice Emery, Daniel Epling, Brandon Smith, Ishan Shah, Maneesha Paranjpe, Shiron Lee, Ching‐Lien Fang, Alison Walsh, Matteo Placidi, Krishanu Mathur, John Mondick, Sunny Chapel, Bernardino Ghetti, Todd Carter, Steven M. Paul, Johnny Yao, Dinah. W.Y Sah

**Affiliations:** ^1^ Voyager Therapeutics, Cambridge, MA USA; ^2^ Akamai Clin Pharm Consulting, Inc., Kula, HI USA; ^3^ A2‐Ai LLC, Ann Arbor, MI USA; ^4^ Department of Pathology and Laboratory Medicine, Indiana University School of Medicine, Indianapolis, IN USA; ^5^ Washington University School of Medicine in St Louis, St Louis, MO USA

## Abstract

**Background:**

VY‐TAU01 is a recombinant humanized IgG4 monoclonal antibody (mAb) directed against pathological tau for the treatment of patients with mild dementia or mild cognitive impairment due to Alzheimer’s disease (AD). Both VY‐TAU01 and its parental mouse IgG1 mAb Ab‐01 target an epitope in the C‐terminus of tau, bind pathological tau with high affinity and selectivity over wild‐type tau, block paired helical filament seed‐induced tau aggregates in vitro, and selectively stain tau tangles in AD and P301S mouse (C57/B6J‐Tg[Thy1‐MAPT*P301S]2541Godt) brain. Ab‐01 robustly inhibits seeding and propagation of pathological tau in a P301S mouse seeding model.

To support toxicology studies and the initiation of the first‐in‐human study, nonclinical studies have been conducted to characterize the pharmacokinetics (PK) and pharmacodynamics (PD) of Ab‐01 in P301S mice and the PK of VY‐TAU01 in cynomolgus macaques.

**Method:**

The PK of Ab‐01 in the P301S mouse after 5 weekly intravenous or intraperitoneal doses at 10 to 120 mg/kg and VY‐TAU01 in cynomolgus macaques after a single intravenous high or mid dose was evaluated with validated ELISAs using their target epitope peptide. The PD of Ab‐01 in the P301S mouse was also evaluated using an ELISA to quantify unbound p‐tau levels.

**Result:**

Ab‐01 and VY‐TAU01 PK profiles in serum and cerebrospinal fluid (CSF) were characterized by a distribution phase followed by a typical elimination phase without evidence of substantial target‐mediated disposition in the respective compartments. Serum and CSF concentrations increased with increasing dose levels in an approximately dose proportional manner, and their half‐lives were approximately 9 to 13 days. CSF concentrations were 0.1 ‐ 0.2% of serum concentrations.

PD effects of Ab‐01 in the P301S mouse were robust, with up to ∼90% lowering of unbound p‐tau. Additional PD as well as PK/PD modeling results will also be presented.

**Conclusion:**

The PK of Ab‐01 in the P301S mouse and VY‐TAU01 in the cynomolgus macaque, and CSF to serum ratios were typical of murine IgG1 and human IgG4 administered to these respective species. The PD of Ab‐01 in the P301S mouse demonstrated robust lowering of unbound p‐tau. These results support VY‐TAU01’s continued development and advancement into the clinic.

